# Chromosomal Aberrations in *ETV6/RUNX1*-positive Childhood Acute Lymphoblastic Leukemia using 244K Oligonucleotide Array Comparative Genomic Hybridization

**DOI:** 10.1186/1755-8166-5-41

**Published:** 2012-11-15

**Authors:** Zubaidah Zakaria, Mohd Fadly Md Ahid, Azli Ismail, Ten Sew Keoh, Nooraisyah Mohamad Nor, Nor Rizan Kamaluddin, Ezalia Esa, Lam Kah Yuen, Eni Juraida Abdul Rahman, Raudhawati Osman

**Affiliations:** 1 Hematology Unit, Cancer Research Center, Institute for Medical Research, Kuala Lumpur, 50588, Malaysia; 2 Pediatrics Institute, Kuala Lumpur Hospital, Kuala Lumpur, 50588, Malaysia; 3 Department of Pathology, Kuala Lumpur Hospital, Kuala Lumpur, 50588, Malaysia

**Keywords:** Array-based Comparative Genomic Hybridization, Acute lymphoblastic leukemia, *ETV6/RUNX1*

## Abstract

**Background:**

Acute lymphoblastic leukemia (ALL) is a heterogeneous form of hematological cancer consisting of various subtypes. We are interested to study the genetic aberration in precursor B-cell ALL with specific t(12;21) translocation in childhood ALL patients. A high resolution 244K array-based Comparative Genomic Hybridization (array-CGH) was used to study eleven *ETV6/RUNX1*-positive childhood acute lymphoblastic leukemia (ALL) patients.

**Result:**

155 chromosomal aberrations (119 losses, 36 gains) were reported in the array findings, corresponding to 76.8% deletions and 23.2% amplifications. The *ETV6* gene deletion occurred in 4 of the patients, corresponding to 45% of the sample. The most common alterations above 1 Mb were deletion 6q (13%), 12p (12%) and 9p (8%), and duplication 4q (6%) and Xq (4%). Other genes important in ALL were also identified in this study including *RUNX1, CDKN2A, FHIT*, and *PAX5*. The array-CGH technique was able to detect microdeletion as small as 400 bp.

**Conclusion:**

The results demonstrate the usefulness of high resolution array-CGH as a complementary tool in the investigation of ALL.

## Background

In childhood B-cell precursor acute lymphoblastic leukemia (ALL), t(12;21)(p13;q22) translocation is the most common chromosomal abnormality and occurs in 20-25% of the cases [1]. This reciprocal translocation, which has a favorable prognosis of more than 80%, leads to the formation of the *ETV6/RUNX1* (also known as *TEL/AML1*) fusion gene [1,2]. The *ETV6/RUNX1* fusion gene was reported in 19% of the Malaysian childhood ALL [3].

Based on the primary *ETV6/RUNX1* leukemogenic model, the translocation arises in utero and the rearrangement alone is not sufficient to develop the disease itself [4]. Other secondary genetic alterations (or "hits") are required to trigger the disease progression; however the role of the additional aberrations has not been fully determined [4]. The secondary events such as copy number alterations and point mutations have been suggested to occur postnatally [5]. Major gene targets that are affected in the *ETV6/RUNX1* fusion involve genes for stem cell development or lineage specification in hematopoiesis [6]. Such fusion in B-cell ALL leads to the activation of kinase or alteration of transcriptional regulations [6].

Various techniques to screen and study chromosomal aberrations in ALL have been developed in recent years. Conventional cytogenetics is routinely used in the initial assessment for the purpose of classification of specific leukemia [7]. Complementary techniques to conventional cytogenetics such as fluorescence in situ hybridization (FISH), and reverse transcriptase - polymerase chain reaction (RT-PCR) can be used as screening tools for *ETV6/RUNX1*-positive patients [8]. FISH study is useful to identify specific translocation, but it is limited to the type of probe used to bind the genomic region of interest, and is not genome-wide [9]. Array comparative genomic hybridization (array CGH) has been applied to study copy number alterations and genomic imbalances for evaluation of patients with ALL [10]. Pathogenic chromosomal abnormalities have been reported in patients using the array-based platform, suggesting the usefulness of this technique for diagnostic services.

In the present study, eleven *ETV6/RUNX1*-positive childhood ALL patients confirmed by RT-PCR were investigated using high resolution array-based comparative genomic hybridization (Agilent 244K Human Genome CGH Microarray).

## Results

### Array CGH

Based on the array-CGH data, a total of 155 genomic aberrations (36 gains, 119 losses, excluding copy number polymorphisms) were identified in all eleven patients, including a patient (no. 4) with duplication of whole chromosome 16. The aberrations ranged from 400 bp to 91.2 Mb. The number of aberrations per patient ranged from 3 to 58, with mean of 14 aberrations per patient. Of the 119 deletions detected, 39 were above 1 Mb and 80 were below 1 Mb. Of the 36 gains detected, 12 were above 1 Mb and 24 were below 1 Mb. In agreement with previous report, we found more deletions (76.8%) than amplifications (23.2%) [11]. Five out of 11 patients (45%) have a deletion of *ETV6* gene. The detected aberrations included previously reported loss/gains that are related to ALL, such as 9p13.2 loss involving *PAX5* in patient no. 2 [12]; 9p21.3 loss involving *CDKN2A* in 4 patients (nos. 3, 4, 6 and 7) and *MLLT3* in patient no. 3 and 4. Only one patient (no. 11) showed a 0.05 Mb deletion on *RUNX1* gene. Three patients (nos. 8, 9 and 10) showed no gross genomic imbalances.

As summarized in Table 1, chromosome 2, 11, 16, 17, 19, 20, 21, and 22 did not have any aberrations above 1 Mb. The most common alterations above 1 Mb were deletion 6q (13%), 12p (12%) and 9p (8%), and amplification 4q (6%) and Xq (4%). The gene annotations are according to the University of California Santa Cruz Genome Browser on Human March 2006 Assembly (NCBI36/hg18).

**Table 1:** Aberrations in 11 patients >1 Mb based on array-CGH findings

**Case No.**	**Gain**	**Loss**	**Size (Mb)**
1	amp(4)(q31.1)		1.2
	amp(4)(q31.3)		3.1
	amp(4)(q35.1-q35.2)		4.2
	amp(18)(q11.2-q23)		58.5
		del(5)(q13.2)	1.8
		del(12)(q13.33)	19.4
2		del(6)(q26-q27)	1.7
		del(12)(p13.2-p11.2)	16.6
3	amp(15)(q11.2)		1.4
	amp(X)(q28)		2.1
		del(12)(p13.2-p13.1)	3.7
4	amp(8)(q23.3-q24.3)		32.2
		del(30(q11.2-q12.3)	5.1
		del(6)(p21.31-p21.2)	1.0
		del(6)(q14.1-q27)	90.8
		del(6)(p25.1-q26)	13.1
		del(9)(p24.2-p24.1)	4.2
		del(9)(p22.1 - p21.3)	2.4
		del(14)(q22.2 - q23.1)	4.2
		del(14)(q24.1 - q24.3)	6.4
		del(15)q11.2	1.3
5		del(1)q31.1 - q31.2	4.0
		del(1)q31.3	2.4
		del(1)q42.2 - q43	4.8
		del(3)p25.1 - p24.3	3.1
		del(3)q26.1	5.6
		del(4)q32.3	2.2
		del(5)q21.1 - q23.3	26.1
		del(6)p22.1 - p21.33	2.8
		del(6)q15 - q22.2	27.6
		del(6)q25.2 - q26	11.2
		del(6)q25.3	2.5
		del(8)q21.13 - q21.2	2.4
		del(8)q23.2 - q23.3	4.9
		del(12)p13.2 - p12.3	4.4
		del(12)q21.1	2.9
		del(12)q21.31 - q21.32	3.2
		del(13)q21.31 - q21.33	3.1
		del(13)q21.31 - q21.33	10.6
		del(13)q31.1 - q31.3	9.1
6	amp(X)q11.1 - q28		91.0
		del(6)q14.1 - q27	91.2
		del(9)p21.3	1.5
7	amp(10)q11.21 - q11.22		2.4
	amp(15)q11.2		1.3
		del(9)p21.3	3.1
8	No gross imbalances		
9	amp(X)p22.31		1.6
10	No gross imbalances		
11		del(7)q31.1-q31.32	15.8
		del(13)q14.2-q21.33	24.9
		del(Y)q11.221-q11.23	10.1

### FISH

**Figure 1:**
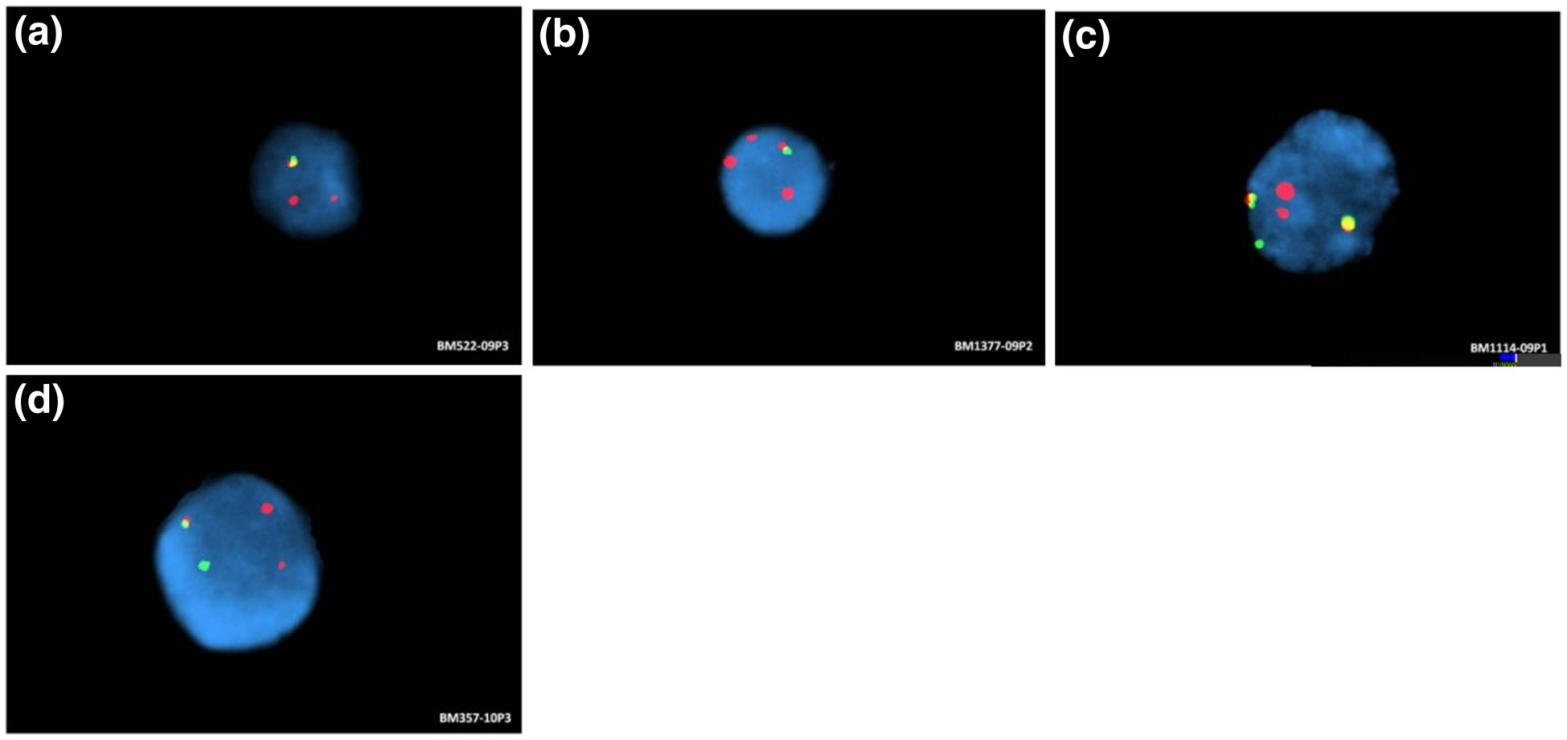
**FISH in 4 patients.** (**a**) Case no. 1 shows 1 red,1 residual red and 1 fusion signal, indicative of loss of *ETV6*; (**b**) Case no. 3 shows 3 red and 1 fusion signal, indicative of extra RUNX1 and loss of *ETV6*; (**c**) Case no. 6 shows 2 fusion,1 red,1 residual red and 1 green signal; (**d**) Case no. 9 shows a typical FISH profile for t(12:21).

All five patients (nos. 1, 3, 5, 6 and 9) showed positive fusion signals of *ETV6/RUNX1*. Two patients (nos. 1 and 5) showed one fusion signal, but no green signal, indicating a loss of *ETV6* gene (Figure 1). Patient no.3 showed a single fusion signal and three red signals, indicating a duplication of *RUNX1* and a loss of *ETV6* gene in 55% interphase cells scored (Figure 1). Interestingly, two fusion signals were identified in patient no. 6. Patient no. 9 showed a typical fusion profile for t(12;21) translocation (Figure 1).

The status of the *ETV6* and *RUNX1* genes in the patients based on array-CGH and FISH findings are as shown in Table 2.

**Table 2:** Array-CGH and FISH findings in patients

**Case No.**	**Array CGH result**	**Significant genes affected**	**FISH result**
1	Loss of 12p13 (*ETV6*), no loss on *RUNX1*	Loss of *ETV6*	Rr, 1F - 92%
2	Loss of 12p13 (*ETV6*); no gain on *RUNX1*	Loss of *PAX5*, *ETV6*, *KRAS*	NA
3	Loss of 12p13 (*ETV6*); no gain on *RUNX1*	Loss of *MLLT3*, *CDKN2A*/B, *MTAP*, *NOTCH1, BTG1, ETV6*	3R, 1F - 55% 2R, 1F - 39%
4	Loss of *ETV6* or *RUNX1* was not found	Loss of *MYB, JAK2*, *MLLT3*, *CDKN2A*/B, *MTAP*	NA
5	Loss of 12p13 (*ETV6*); no loss on *RUNX1*	Loss of *ETV6*	Rr, 1F - 95%
6	Loss of *ETV6* or *RUNX1* was not found	Loss of MYB, *MTAP*, *CDKN2A*/B,	2F, Rr, 1G - 60% Rr, 1F, 1G - 37%
7	Loss of 12p13 (*ETV6*); no gain on *RUNX1*	Loss of *MTAP*, *CDKN2A*/B, *ETV6*, *FHIT*	NA
8	Loss of *ETV6* or *RUNX1* was not found	Appears normal	NA
9	Loss of *ETV6* or *RUNX1* was not found	Appears normal	Rr, 1G, 1F - 100%
10	Loss of *ETV6* or *RUNX1* was not found	Appears normal	NA
11	Loss of 21q22 (*RUNX1*); no loss on *ETV6*	Loss of *RUNX1*	NA

F = Fusion (*ETV6-RUNX1* fusion); R = Red (normal *RUNX1* gene); r = residual red (residual *RUNX1*gene disrupted by the translocation); G = Green (*ETV6* gene).

## Discussion

Our data have demonstrated that the 244K oligonucleotide array-CGH platform is a powerful tool to detect additional copy number alterations in *ETV6/RUNX1*-positive patients. A total of 155 aberrations were identified, including microdeletions as small as 400 bp. Many known or potential genes related to leukemia were also identified using this method. These data supported the secondary leukemogenic model that additional aberrations are necessary for leukemogenesis. According to our array data, 5 out of 11 patients (45%) showed deletion involving *ETV6* gene from as small as 0.2 Mb to 19.4 Mb. We found more deletions (76.8%) than amplifications (23.2%), which is in agreement with a previous study [11]. Among the deletions, 32.7% were larger than 1 Mb, while 33.3% of the amplifications were larger than 1 Mb.

Patient no. 2 harbored a 0.09 Mb deletion on 9p13.2 that involved the *PAX5* gene. *PAX5* is important in the normal development of B cells, in which loss of a wild-type *PAX5* allele would cause differentiation arrest in ALL [12]. Deletion of the tumor suppressor *CDKN2A* gene located at 9p21.3 was found in 36% (4/11) of our patients. The *CDKN2A* deletion is suggested to occur more frequently in T-ALL than in precursor B-ALL [13]. The deletion is thought to vary by cytogenetic subgroup and the prognostic value of the incidence is yet to be determined [14]. One patient (no. 7) was found to have a gross deletion (1.0 Mb) on 3p14.2 region that included the *FHIT* gene, which is proposed as a putative tumor-suppressor gene. The deletion on this particular gene was found to be correlated with a low clinical remission rate and poor overall survival [15-17].

Several putative target genes within the commonly gained region, including cryptic Xq duplications were also found in patient no. 3 and 6, both females. The sizes of the gains on the two patients were 2.1 Mb and 91 Mb, respectively. This result is discordant with the previous report that males are more common to harbor this aberration [18]. This discrepancy may be explained by the small sample size used in this study. It would be interesting to study the expression level of *ETV6/RUNX1* proposed genes, namely the SPANX family genes, on the X chromosome in our female's dataset.

Based on our FISH study on five childhood ALL patients, all samples showed a positive *ETV6/RUNX1* fusion signal. Three patients (nos. 1, 3 and 5) showed concordant result with array CGH for *ETV6* gene deletion. FISH result for patient no. 3 showed three red signals, suggesting that there was a duplication of the *RUNX1* signal, but was not confirmed through the array findings. It has been reported that DNA microarray may fail to detect the chromosomal abnormalities if the abnormal clones are present in fewer than 25% of the cell population [19].

Patient no. 6 showed a unique FISH profile where two fusion signals of the *ETV6/RUNX1* were detected. Double *ETV6/RUNX1* fusion signals were found in 25% of *ETV6/RUNX1* positive ALL patients [20]. Previous studies have found that the additional *ETV6/RUNX1* fusion signal may have arisen from duplication of the der(21)t(12;21) chromosome [21,22], duplication of *ETV6/RUNX1* fusion gene that was later translocated onto another chromosome [22] or ider(21)(q10)t(12;21)(p12;q22) [23]. In the study by Loncarevic and coworkers (1999), gain of the der(21)t(12;21) chromosome was found exclusively in the relapsed cases [21]. We were not able to ascertain the origin of the extra *ETV6/RUNX1* fusion signal in our patient due to non-availability of metaphase cytogenetics. It has however been suggested that secondary changes such as the duplication of fusion signals may contribute to the process of leukemogenesis [22].

Three of the patients, namely patient nos. 4, 10 and 11, had a relapse. Of the three, patient no. 4 had multiple gross deletions as large as 90.8 Mb, whereas patient no. 11 had other gross imbalances larger than 1 Mb. However, the array report for patient no. 10 showed no gross imbalances larger than 1 Mb. We could not determine whether any subsequent aberrations happened after the sample was taken which might trigger the relapse event.

## Conclusion

Our study indicates that high resolution oligonucleotide array-CGH is an essential complementary tool in the investigation of the *ETV6/RUNX1* positive ALL patients as it helps to complement the findings of FISH and RT-PCR as well as overcoming the limitations of conventional cytogenetics which require cell culture and quality metaphases for analysis. Indeed, array-CGH has revealed additional aberrations which may have pathogenetic implications. A larger cohort is however needed to comprehensively study the genetic diversity of our *ETV6/RUNX1*- positive ALL cases.

## Materials and methods

### Patients

Eleven *ETV6/RUNX1*-positive childhood ALL patients (7 boys and 4 girls) with ages ranging from 2 to 11 years old were selected for this study. All patients were diagnosed as precursor B-ALL with CALLA positivity based on their immuno-phenotyping report. The presence of *ETV6/RUNX1* fusion gene in all cases was ascertained by HemaVision® Multiplex RT-PCR System (Bio-Rad Laboratories, Hercules, CA) as part of the routine diagnostic procedure. The study was approved by the Medical Research & Ethics Committee, Ministry of Health Malaysia.

DNAs were extracted from bone marrow aspirates using QIAGEN DNAeasy Blood Kit (Qiagen, Hilden, Germany) according to manufacturer’s instruction. DNA samples subjected to array CGH were of sufficient quality with A260/280 ratio >1.8 as measured by NanoDrop ND-1000 UV–VIS spectrophotometer.

### Array-CGH

Array-CGH analysis on the 11 patient samples was carried out using Human Genome CGH 244A Microarray Kit (Agilent Technologies, CA, USA) according to the manufacturer’s protocols. In brief, 1–3 μg of DNA from each patient and reference sample was subjected to restriction digestion using AluI and RsaI restriction enzymes. The reference DNA was commercially obtained from Promega (Promega, Madison, WI) and was gender-matched accordingly. The completion of the digestion for each sample was analyzed using Bioanalyzer before the samples were labeled using Cy3 and Cy5 for patient samples and reference samples, respectively. The yield, the degree of labeling, and the specific activity of the samples were measured using NanoDrop before the two respective samples were combined. The hybridization procedure was carried out at 37 °C for 40 hours and the slides were scanned using Agilent DNA Microarray Scanner. The images from the array-CGH were processed using Agilent Feature Extraction Software (version 9.5.31). The data obtained were analyzed using DNA Analytics v4.0.76 software (Agilent Technologies) with an ADM-2 algorithm with threshold 6.0, and minimum adjacent 3 probes required to be gained or lost for a call to be made. The array-CGH data for all samples have been deposited in Gene Expression Omnibus and are accessible through GEO series accession number GSE32897.

### FISH

Five patients with available suspension were selected for FISH studies. The slides for FISH analysis were prepared using cell suspension and were dried at 60°C overnight. The Vysis LSI *ETV6/RUNX1* ES dual-color probe was used to identify the translocation pattern for these samples. The probe was added to the slides, hybridized on HyBrite and washed through a series of washes. The slides were viewed under fluorescent microscope, and at least 200 interphase nuclei were analysed for their fusion signal.

## Competing interest

All authors declare no competing interest.

## Author’s contribution

ZZ designed the experimental study and drafted the manuscript. MFMA and AI carried out the array CGH experiments and performed the data analysis. TSK participated in the FISH analysis and helped draft the manuscript. NMN carried out additional array CGH experiments, performed data analysis and helped draft the manuscript. NRK and EE participated in the design study. EJAR and RO provided the clinical details of the patients, LKY performed FISH analysis. All authors read and approved the final manuscript.
